# Early Detection, Curative Treatment, and Survival Rates for Hepatocellular Carcinoma Surveillance in Patients with Cirrhosis: A Meta-analysis

**DOI:** 10.1371/journal.pmed.1001624

**Published:** 2014-04-01

**Authors:** Amit G. Singal, Anjana Pillai, Jasmin Tiro

**Affiliations:** 1 Department of Internal Medicine, University of Texas Southwestern Medical Center, Dallas, Texas, United States of America; 2 Department of Clinical Sciences, University of Texas Southwestern, Dallas, Texas, United States of America; 3 Harold C. Simmons Cancer Center, University of Texas Southwestern Medical Center, Dallas, Texas, United States of America; 4 Department of Internal Medicine, Emory University, Atlanta, Georgia, United States of America; University of Oxford, United Kingdom

## Abstract

**Background:**

Surveillance for hepatocellular carcinoma (HCC) has level I evidence among patients with hepatitis B but only level II evidence in patients with cirrhosis. This lack of randomized data has spurred questions regarding the utility of HCC surveillance in this patient population; however, lack of randomized data does not equate to a lack of data supporting the efficacy of surveillance. The aim of our study was to determine the effect of HCC surveillance on early stage tumor detection, receipt of curative therapy, and overall survival in patients with cirrhosis.

**Methods and Findings:**

We performed a systematic literature review using Medline from January 1990 through January 2014 and a search of national meeting abstracts from 2009–2012. Two investigators identified studies that reported rates of early stage tumor detection, curative treatment receipt, or survival, stratified by HCC surveillance status, among patients with cirrhosis. Both investigators independently extracted data on patient populations, study methods, and results using standardized forms. Pooled odds ratios, according to HCC surveillance status, were calculated for each outcome using the DerSimonian and Laird method for a random effects model.

We identified 47 studies with 15,158 patients, of whom 6,284 (41.4%) had HCC detected by surveillance. HCC surveillance was associated with improved early stage detection (odds ratio [OR] 2.08, 95% CI 1.80–2.37) and curative treatment rates (OR 2.24, 95% CI 1.99–2.52). HCC surveillance was associated with significantly prolonged survival (OR 1.90, 95% CI 1.67–2.17), which remained significant in the subset of studies adjusting for lead-time bias. Limitations of current data included many studies having insufficient duration of follow-up to assess survival and the majority not adjusting for liver function or lead-time bias.

**Conclusions:**

HCC surveillance is associated with significant improvements in early tumor detection, receipt of curative therapy, and overall survival in patients with cirrhosis.

*Please see later in the article for the Editors' Summary*

## Abstract 2

Amit Singal and colleagues conducted a systematic review of the evidence that surveillance for hepatocellular carcinoma in patients with cirrhosis improves early detection, receipt of curative treatment, and overall survival.

*Please see later in the article for the Editors' Summary*

## Editors' Summary

**Background:**

Hepatocellular cancer (HCC) is the commonest form of primary liver cancer—a type of cancer that starts when a cell in the liver acquires genetic changes that allow it to grow uncontrollably. Primary liver cancer is the third leading cause of cancer-related death worldwide, killing more than 600,000 people every year. The symptoms of HCC are vague and rarely appear until the cancer has spread throughout the liver. They include unexplained weight loss, feeling sick, tiredness, and jaundice (yellowing of the skin and eyes). If liver cancer is diagnosed in its early stages, it can be treated by surgically removing part of the liver, by liver transplantation, or by a procedure called radiofrequency ablation in which an electric current is used to destroy the cancer cells. However, most people are diagnosed with HCC when the cancer is advanced and cannot be treated. These individuals are given palliative treatment to relieve pain and discomfort. Although most patients who are diagnosed with HCC at an early stage survive more than 5 years, patients with more advanced HCC have an average survival less than one year. The exact cause of HCC is unknown, but it is thought to be related to cirrhosis (scarring) of the liver. This condition is the end result of long-term (chronic) liver damage caused by, for example, alcohol abuse or infection with hepatitis B virus (HBV).

**Why Was This Study Done?:**

Because HCC tends to be untreatable when it is diagnosed at a late stage, if the tumor can be found early by regularly measuring blood levels of alpha fetoprotein (a liver cancer biomarker) and using ultrasound, outcomes for patients at high risk of developing HCC might be improved. Indeed, American and European guidelines recommend HCC surveillance with ultrasound every 6 months in patients with HBV infection and/or cirrhosis. However, although randomized controlled trial results support HCC surveillance among patients infected with HBV, no randomized trials have investigated its use among patients with cirrhosis. Here, the researchers use predefined criteria to identify all the published cohort and case-control studies (two types of non-randomized studies) that have examined the impact of HCC surveillance on outcomes in patients with cirrhosis. They then pool the data from these studies using a statistical approach called meta-analysis to estimate whether HCC surveillance is associated with improvements in early tumor detection, curative treatment receipt, and survival rates among patients with cirrhosis.

**What Did the Researchers Do and Find?:**

The researchers identified 47 studies that examined the association of HCC surveillance with outcomes in 15,158 patients with cirrhosis who developed HCC. In 41.4% of these patients, HCC was detected by surveillance. Among patients who had undergone HCC surveillance, the pooled rate of early detection was 70.9%, whereas among patients who had not undergone surveillance but who were diagnosed incidentally or who presented with symptoms, the pooled rate of early detection was 29.9%. The researchers calculated that the pooled odds (chances) of early detection among patients undergoing surveillance compared to early detection among patients not undergoing surveillance was 2.08 (an odds ratio [OR] of 2.08). The pooled rate of curative treatment receipt among patients undergoing surveillance was 51.3% compared to only 23.8% among patients not undergoing surveillance (OR 2.24). Finally, among those patients for whom the relevant data were available, 50.8% of patients who had undergone HCC surveillance but only 28.2% of those who had not undergone surveillance survived for at least 3 years after diagnosis (OR 1.90).

**What Do These Findings Mean?:**

These findings show that HCC surveillance is associated with significant improvements (improvements that are unlikely to have happened by chance) in early tumor detection, receipt of curative treatment, and overall survival among patients with cirrhosis. Importantly, the association with improved overall survival remained significant after adjusting for the possibility that patients who underwent surveillance died at the same time as they would have done without surveillance but appeared to survive longer because they were diagnosed earlier (this is called adjustment for lead-time bias). These results must be interpreted cautiously, however, because many of the studies included in the meta-analysis had insufficient follow-up to assess survival adequately, not all the studies adjusted for lead-time bias, and none of the studies assessed potential downstream harms of HCC surveillance such as complications of liver biopsies. Nevertheless, overall, these findings provide sufficient evidence to support guidelines that recommend regular HCC surveillance for patients with cirrhosis.

**Additional Information:**

Please access these websites via the online version of this summary at http://dx.doi.org/10.1371/journal.pmed.1001624.

- The US National Cancer Institute provides information about all aspects of cancer, including detailed information for patients and professionals about primary liver cancer and about screening for primary liver cancer (in English and Spanish)
- The American Cancer Society also provides information about liver cancer (available in several languages)
- The UK National Health Service Choices website provides information about primary liver cancer and about cirrhosis (including patient stories)
- Cancer Research UK (a not-for-profit organization) also provides detailed information about primary liver cancer
- MedlinePlus provides links to further resources about liver cancer and cirrhosis (in English and Spanish)
- Information is available at the American Liver Foundation
- American Association for the Study of Liver Diseases provides practice guidelines

## Introduction

Hepatocellular carcinoma (HCC) is the third leading cause of cancer-related death worldwide and one of the leading causes of death among patients with cirrhosis [1]. Its incidence in the United States and Europe is increasing due to the current epidemic of nonalcoholic steatohepatitis (NASH) and hepatitis C virus (HCV) cases [2]. Prognosis for patients with HCC depends on tumor stage, with curative therapies only available for patients detected at an early stage. Patients detected at an early stage can achieve 5-year survival rates of 70% with transplant or resection, whereas those with advanced HCC are only eligible for palliative treatments and have a median survival of less than one year [3],[4].

The American Association for the Study of Liver Diseases (AASLD) and European Association for the Study of the Liver (EASL) guidelines recommend surveillance with ultrasound every 6 months in high-risk patients, i.e., those with chronic hepatitis B virus (HBV) infection and/or cirrhosis [5],[6]. The goal of surveillance is to detect HCC at an early stage when it is amenable to curative therapy and to reduce all cause mortality. Although surveillance among HBV patients is supported by a large randomized controlled trial, there is no similar level I evidence supporting this practice among patients with cirrhosis [7]. Data from patients with HBV cannot be directly extrapolated to patients with cirrhosis for several reasons, including a higher competing risk of non-HCC mortality and lower sensitivity of surveillance tools for HCC with a nodular liver [8]. The lack of randomized data has spurred questions regarding the utility of HCC surveillance in this patient population [9].

Given the lack of a randomized trial of HCC surveillance among patients with cirrhosis, a meta-analysis of cohort and case-control studies can serve to better characterize any potential benefits of HCC surveillance. The aim of our study was to determine the association of HCC surveillance with (i) detection of tumors at an early stage, (ii) receipt of curative therapies, and (iii) overall survival in patients with cirrhosis.

## Methods

### Data Sources and Searches

We conducted a computer-assisted search with the Ovid interface to Medline to identify relevant published articles. We search the Medline database from January 1, 1989 through January 1, 2014 with the following keyword combinations: (liver ca$ OR hepatocellular ca$ OR hcc OR hepatoma) AND (screen$ OR surveillance OR ultrasound). We chose to include studies after January 1989 to accurately reflect the current performance of ultrasonography and the current availability of curative therapies (including liver transplantation and radiofrequency ablation [RFA]). Manual searches of reference lists from applicable studies were performed to identify any studies that may have been missed by the computer-assisted search. Additional searches of AASLD, EASL, Digestive Diseases Week (DDW), American College of Gastroenterology (ACG), and American Society of Clinical Oncology (ASCO) meeting abstracts from 2010–2012 were performed. Finally, consultation with expert hepatologists was performed to identify additional references or unpublished data. This study was conducted in accordance with PRISMA guidelines [10].

### Study Selection

Two investigators (AGS and AP) reviewed citations identified by the search strategy to generate a list of potentially relevant articles. The abstract for each potentially relevant study was then reviewed by each of the two investigators. If the applicability of a study could not be determined by title or abstract alone, the full text was reviewed. Articles were independently checked for possible inclusion and disagreements were resolved through consensus with a third reviewer (JT).

Studies were included for analysis if they (i) utilized ultrasound, with or without concomitant alpha fetoprotein (AFP), for HCC surveillance; (ii) performed surveillance in a cohort of patients with cirrhosis; and (iii) reported the number of HCC detected at an early stage, number of HCC patients who received curative therapies, and/or overall survival in both patients undergoing surveillance and those not undergoing surveillance. If a study included both patients with cirrhosis and chronic hepatitis, only data regarding patients with cirrhosis were extracted if possible. We included articles published in English or Spanish. We excluded studies that (i) evaluated one-time screening instead of surveillance or (ii) only reported outcome measures for patients undergoing surveillance but not for those without surveillance. Additional exclusion criteria included non-human data, lack of original data and incomplete reports. If duplicate publications used the same cohort of patients, data from the most recent article were included.

### Data Extraction and Quality Assessment

Two reviewers (AGS and AP) independently extracted required information from eligible studies using standardized forms. A third investigator (JT) was available to resolve discrepancies between the two sets of extracted data. The data extraction form included the following study design items: characteristics and size of study cohort, inclusion and exclusion criteria, surveillance tests, surveillance interval, and definition of early stage disease. In addition, we recorded the following primary data for patients who received and did not receive surveillance: number of patients with HCC, proportion of HCC discovered at an early stage, proportion of patients who received curative treatments, and overall survival. Two investigators (AGS and AP) assessed study quality by a modified checklist based upon the Ottawa-Newcastle scale (ONS), with discrepancies resolved by consensus. This instrument rates observational studies on a nine-point scale based on appropriateness of study sample, comparability of study groups, and adequacy of assessing exposure and outcomes [11].

### Data Synthesis and Statistical Analysis

For each individual study, an odds ratio for each outcome of interest was calculated according to receipt of surveillance (i.e., surveillance group versus non-surveillance group). Our first outcome of interest was the proportion of patients diagnosed with early stage HCC. Early stage HCC was defined by Milan criteria, i.e., one tumor less than 5 cm in maximum diameter or two to three lesions, each with a maximum diameter less than 3 cm [12]. Insufficient data on performance status and liver function in most studies precluded use of the Barcelona Clinic Liver Cancer (BCLC) staging system. The second outcome of interest was the proportion of patients with HCC who underwent curative therapy. Curative treatments included any of the following: liver transplantation, surgical resection, RFA, or percurateous ethanol injection (PEI). Although transarterial chemoembolization (TACE) has been demonstrated to improve survival, it is regarded as palliative and was not included in our treatment outcome. Finally, our third outcome of interest was overall survival.

For each outcome of interest, we calculated a pooled odds ratio estimate with corresponding 95% confidence intervals, using the DerSimonian and Laird method for a random effects model. Heterogeneity was evaluated graphically by examination of forest plots and then statistically by the chi-squared test of heterogeneity and the inconsistency index (I^2^). A chi-squared *p*-value<0.05 or I^2^ values >50% are consistent with possible substantial heterogeneity [13],[14]. Meta-influence analysis, in which one study is removed at a time, was performed to determine if there was possible undue influence of a single study. Publication bias was evaluated graphically by funnel plot analysis (Figures S1, S2, S3) and then statistically using Begg's test [15]. An asymmetric funnel plot would suggest the possibility of small studies not being published due to unfavorable results.

Subset analyses were planned for predefined variables, including (i) location of study (Asia versus Europe versus United States), (ii) study period (prior to 1990s versus 1990s versus 2000s), (iii) proportion of Child Pugh C cirrhosis (<10% versus ≥10%), (iv) type of surveillance tests (ultrasound versus ultrasound and AFP), and (v) length of surveillance interval (≤6 months versus >6 months). Study location and study period were evaluated given potential differences in available technology over time. Subset analyses were planned for type of surveillance tests and surveillance interval, as both have been previously demonstrated to affect surveillance efficacy [16]. Finally, we included population characteristics, such as Child Pugh class, given that liver function is a known determinant of treatment eligibility and survival [5]. All data analysis was conducted using Stata 11.

## Results

### Literature Search

**Figure 1:**
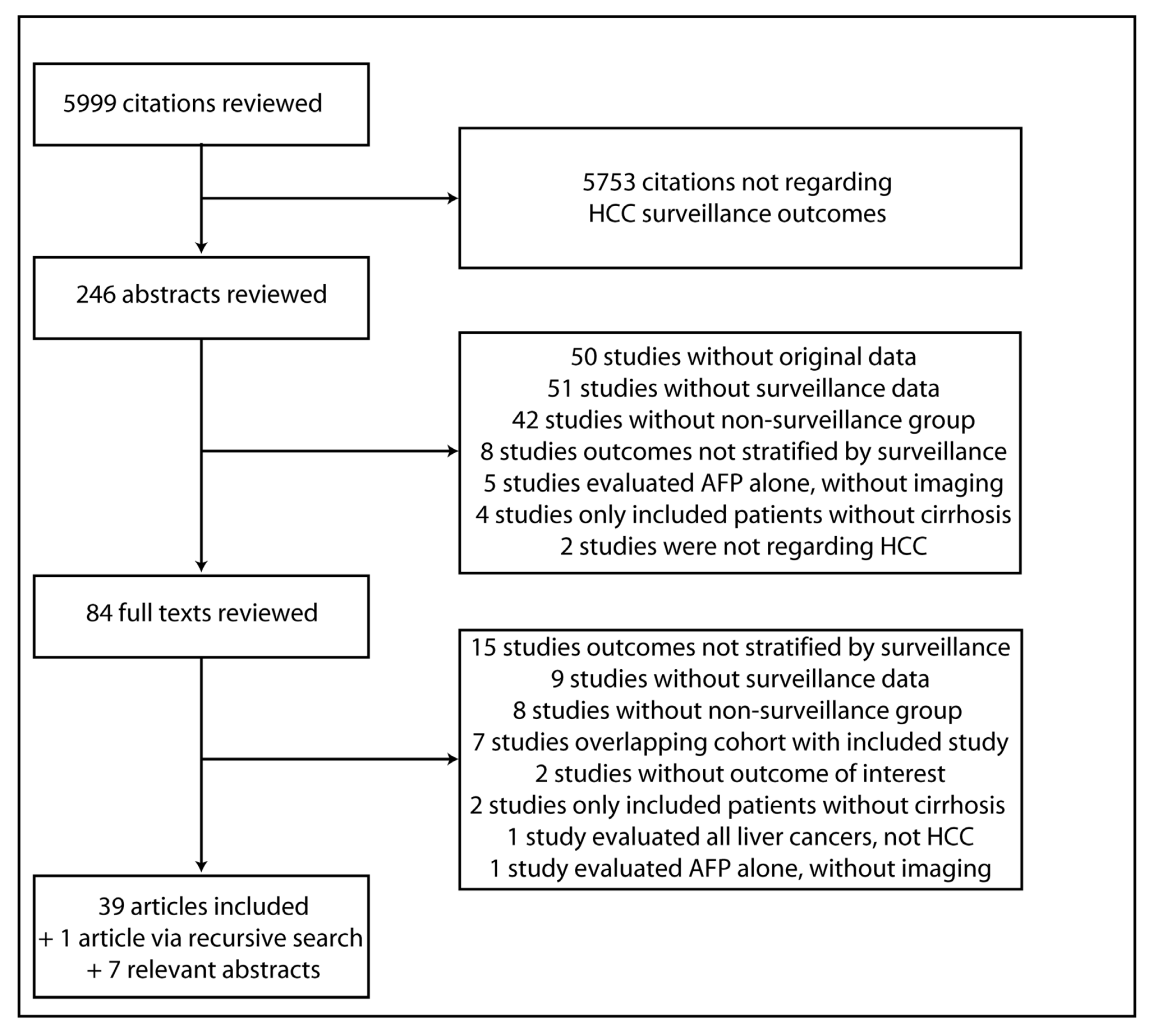
Map of literature search and selection process.

The computer-assisted search yielded 5,999 potentially relevant titles published between January 1, 1989 and January 1, 2014. After initial review, 246 titles were potentially appropriate, and these abstracts were reviewed. Eighty-four publications underwent full-text review, and 45 were excluded. The remaining 39 met all inclusion criteria (Figure 1). Searches of annual meeting abstracts yielded seven relevant abstracts with sufficient data for inclusion. Finally, recursive literature searches identified one additional article that met inclusion criteria, producing a total of 47 studies for inclusion.

On the basis of evaluation of funnel plots (Figures S1, S2, S3), we could not exclude the possibility of publication bias. Most small studies produced larger positive effects than studies with large sample sizes, particularly for receipt of curative therapy and overall survival. There was a paucity of small “negative” studies, i.e., those failing to show a significant association between HCC surveillance and early detection, curative treatment, or overall survival. However, the association between HCC surveillance and each outcome remained statistically significant when only including large studies (i.e., those with at least 100 patients with HCC) (Table 4).

### Study Characteristics

Characteristics of included studies are described in Table 1. We identified 47 studies, with a total of 15,158 patients, assessing the impact of HCC surveillance on at least one outcome of interest [17]–[63]. Of these patients, 6,284 (41.4%) HCC were detected by surveillance and 8,874 (58.6%) presented symptomatically or were found incidentally outside of a surveillance protocol. HCC was detected by surveillance in 51% (1,614 of 3,162) of patients among studies in the United States, 45% (1,182 of 2,611) of patients among studies in Europe, 37% (3,312 of 8,804) of patients among studies in Asia, and 30% (176 of 581) of patients among other studies. Most studies (*n* = 38) were retrospective in nature, although nine had collected data about HCC outcomes prospectively. Fifteen studies were conducted in the United States, 15 in Asia, 13 throughout Europe, and four studies were conducted in other countries. Of the 39 studies that specified surveillance tests used, only ten included ultrasound alone and most used a combination of ultrasound and/or AFP.

**Table 1:** Characteristics of included studies.

AuthorYear	Study Location	Design of Data Collection	Cohort	Surveillance Method	Number of Patients with HCC	Proportion with Child C Cirrhosis	Definition of Early Stage HCC	Proportion of Patients with Early HCC	Proportion of Patients with Curative Treatment	Factors Adjusted for in Survival Analysis	Overall Survival
Singal2013 [45]	United States	Prospective	HCV-associated HCC	US and AFPevery 6 mo	83(72 S, 11 NS)	0%	Milan criteria	72% Surv73% Non surv	NR	N/A	NR
Wong2013 [60]	Australia	Retrospective	HCC	US and AFPevery 6–12 mo	215(70 S, 145 NS)	9.2%	NR	NR	68% Surv30% Non surv	GenderDecade of diagnosisLiver functionAlkaline phosphataseTumor stage	Median 30-mo surv8-mo non surv
Ayala2012 [18]	Lebanon	Retrospective	HCC	Imagingwithin 15 mo	112(54 S, 58 NS)	NR	BCLCStage A	54% Surv46% Non surv	NR	None	72% 1-year surv43% 1-year non surv
Bouali2012 [20]	Tunisia	Retrospective	HCC	USevery 4–12 mo	105(25 S, 80 NS)	NR	Unifocal tumor <3 cm	46% Surv21% Non surv	64% Surv22% Non surv	None	Median 23-mo surv6-mo non surv
Miguel2012 [37]	Spain	Prospective	HCC	US and AFPevery 6 mo	110(56 S, 54 NS)	3.6%	BCLCStage A	71% Surv48% Non surv	71% Surv48% Non surv	Liver functionTumor stageTreatment	Median 32-mo surv21-mo non surv
Sarkar2012 [43]	United States	Retrospective	HBV-associated HCC	US or AFPwithin year	51(14 S, 37 NS)	NR	Milan criteria	79% Surv19% Non surv	71% Surv30% Non surv	AgeGenderCirrhosisLiver functionTumor stageTreatment	67% 3-year surv25% 3-year non surv
El-Serag2011 [27]	United States	Retrospective	HCV-associated HCC	US or AFP within 6 mo and 7–24 mo	912(580 S, 332 NS)	NR	NR	NR	NR	AgeRaceYear of diagnosisLiver functionPsychosisLead time	22% 3-year surv15% 3-year non surv
Kallwitz2011 [33]	United States	Retrospective	HCC	Not defined	167(97 S, 70 NS)	NR	Milan criteria	81% Surv35% Non surv	39% Surv7% Non surv	Tumor stageTreatment	Mortality HR 0.52(95% CI 0.29–0.95)
Reau2011 [41]	United States	Retrospective	HCC	Not defined	110(65 S, 45 NS)	NR	Milan criteria	94% Surv36% Non surv	NR	N/A	NR
Smirniotopoulos2011 [46]	United States	Retrospective	HCC	Imagingwithin year	89(42 S, 47 NS)	NR	TNMStage I–II	98% Surv60% Non surv	52% Surv9% Non surv	N/A	NR
Stroffolini2011 [48]	Italy	Prospective	HCC	US and AFP	411(257 S, 154 NS)	8.6%	Milan criteria	OR 3.1(95% CI 1.9–5.2)	NR	N/A	NR
Yang2011 [61]	United States	Retrospective	HCC	Imagingwithin year	443(136 S, 307 NS)	10.6%	Milan criteria	58% Surv20% Non surv	51% Surv24% Non surv	None	65% 3-year surv16% 3-year surv
Rodriguez2011 [42]	Spain	Prospective	HCC	US and AFP	136(86 S, 50 NS)	5.9%	BCLC A	73% Surv32% Non surv	NR	N/A	NR
Goh2010 [31]	Singapore	Prospective	HCC	US and AFPevery 6–12 mo	1,113(186 S, 927 NS)	NR	TNMStage I–II	59% Surv25% Non surv	51% Surv12% Non surv	None	Median 35-mo surv4-mo non surv
Jou2010 [32]	United States	Retrospective	HCC	Imagingwithin year	319(98 S, 221 NS)	10.0%	BCLCStage A	53% Surv46% Non surv	56% Surv26% Non surv	N/A	NR
Kuo2010 [34]	Taiwan	Retrospective	HCC	US and AFPwithin year	1,436(318 S, 1,118 NS)	6.2%	BCLCStage A	69% Surv27% Non surv	46% Surv23% Non surv	EtiologyLiver functionAFPTumor stageTreatment	59% 3-year surv29% 3-year non surv
Noda2010 [38]	Japan	Retrospective	HCV–associated HCC	Imagingwithin year	240(124 S, 116 NS)	NR	Milan criteria	88% Surv44% Non surv	80% Surv45% Non surv	None	73% 3-year surv52% 3-year non surv
Tong2010 [52]	United States	Retrospective	HCC	US and AFPevery 6–12 mo	278(219 S, 59 NS)	2.9%	Milan criteria	65% Surv25% Non surv	NR	Hepatitis BLiver functionAFPAlkaline phosphataseTumor stage	48% 3-year surv23% 3-year non surv
Tong2010 [53]	United States	Retrospective	HBV–associated HCC	US and AFPwithin year	78(26 S, 52 NS)	5.1%	Milan criteria	62% Surv20% Non surv	50% Surv23% Non surv	Liver functionTumor stageLead time	63% 3-year surv37% 3-year non surv
Zapata2010 [63]	Spain	Retrospective	HCC	US and AFPevery 6 mo	85(40 S, 45 NS)	2.6%	Milan criteria	70% Surv27% Non surv	48% Surv27% Non surv	N/A	NR
Chan2008 [22]	Hong Kong	Prospective	Viral-associated HCC	US and AFPevery 6–12 mo	1,366(441 S, 925 NS)	8.1%	NR	NR	64% Surv36% Non surv	None	62% 3-year surv29% 3-year non surv
Pascual2008 [40]	Spain	Prospective	HCC	US and AFPevery 6 mo	290(117 S, 173 NS)	14.5%	Unifocal tumor <5 cm	60% Surv24% Non surv	47% Surv15% Non surv	Liver functionTumor stageTreatment	46% 3-year surv13% 3-year non surv
Silveira2008 [44]	United States	Retrospective	PBC–associated HCC	US and AFPevery 6–12 mo	33(17 S, 16 NS)	47% hepatic decompensation	Milan criteria	47% Surv56% Non surv	65% Surv50% Non surv	AgeLiver functionTreatment	58% 3-year surv16% 3-year non surv
Stravitz2008 [47]	United States	Retrospective	HCC	Imagingwithin year	279(172 S, 107 NS)	15%	Milan criteria	69% Surv26% Non surv	32% Surv9% Non surv	None	40% 3-year surv19% 3-year non surv
Wong2008 [58]	Hong Kong	Retrospective	Viral-associated HCC	US and AFPevery 6–24 mo	472(79 S, 393 NS)	4.7%	NR	NR	67% Surv30% Non surv	AgeGenderLiver functionLead time	40% 3-year surv20% 3-year non surv
Caumes2007 [21]	France	Prospective	HCC	Not defined	106(30 S, 76 NS)	22.7%	Unifocal tumor <3 cm	33% Surv4% Non surv	37% Surv18% Non surv	N/A	NR
Cho2007 [24]	Korea	Retrospective	HCC	Not defined	71(16 S, 55 NS)	0%	BCLCStage A	65% Surv2% Non surv	NR	AgeGenderCirrhosisViral hepatitisLiver functionTumor stage	Median 60-mo surv16-mo non surv
Davila2007 [25]	United States	Retrospective	HCC	Imaging or AFP within 3 years	157(44 S, 113 NS)	36.3%	Unifocal tumor	50% Surv38% Non surv	NR	None	30% 3-year surv21% 3-year non surv
Gellert2007 [29]	Australia	Retrospective	HCC	US or AFP	149(27 S, 122 NS)	14.1%	Milan criteria	44% Surv20% Non surv	19% Surv10% Non surv	Liver functionTumor sizeTreatment	Median 13-mo surv4-mo non surv
Leykum2007 [35]	United States	Retrospective	HCV–associated HCC	Imaging or AFP within year	72(16 S, 56 NS)	NR	Milan criteria	100% Surv21% Non surv	63% Surv11% Non surv	Tertiary careSubspecialty carePsychosisTumor stageTreatment	30% 3-year Surv15% 3-year non surv
Ando2006 [17]	Japan	Retrospective	HCC	Imaging and AFP	574(392 S, 182 NS)	NR	Milan criteria	73% Surv26% Non surv	57% Surv26% Non surv	None	62% 3-year surv38% 3-year non surv
Cheung2006 [23]	Hong Kong	Retrospective	HCC	US and AFP	223(97 S, 126 NS)	23.3%	TNMStage I–II	47% Surv21% Non surv	NR	Hepatitis BSmokingAlcoholLiver functionAlkaline phosphataseTumor stageTreatment	Median 21-mo surv4-mo non surv
Tanaka2006 [49]	Japan	Retrospective	HCV-related HCC	US and AFPevery 6 mo	384(182 S, 202 NS)	2.6%	Milan criteria	86% Surv50% Non surv	76% Surv46% Non surv	Liver functionAFPTumor stageLead time	67% 3-year surv51% 3-year non surv
Toyoda2006 [54]	Japan	Retrospective	HCC	Imaging or AFP	1,641(1,050 S, 591 NS)	15.1%	TNMStage I–II	58% Surv20% Non surv	44% Surv14% Non surv	AgeGenderLiver functionTumor stageTreatment	51% 3-year surv27% 3-year non surv
Taura2005 [51]	Japan	Retrospective	HCC	US and AFPevery 3–12 mo	271(178 S, 93 NS)	5.9%	NR	NR	51% Surv20% Non surv	Liver function	67% 3-year surv53% 3-year non surv
Van Vlierberghe2005 [57]	Belgium	Prospective	HCC	Not defined	131(47 S, 84 NS)	NR	Milan criteria	60% Surv31% Non surv	NR	None	58% 1-year surv26% 1-year non surv
Yu2004 [62]	Taiwan	Retrospective	HCC	Routine US	680(164 S, 516 NS)	NR	NR	NR	51% Surv29% Non surv	AgeCirrhosisViral hepatitisLiver functionAFPLead time	49% 3-year surv41% 3-year non surv
Trevisani2002 [55]	Italy	Retrospective	HCC	US and AFPevery 6–12 mo	821(370 S, 451 NS)	8.9%	Milan criteria	65% Surv31% Non surv	41% Surv27% Non surv	GenderHepatitis BLiver functionAFPTumor stageTreatmentLead time	48% 3-year surv23% 3-year non surv
Bolondi2001 [19]	Italy	Retrospective	HCC	US and AFPevery 6 mo	165(61 S, 104 NS)	12.1%	NR	NR	48% Surv32% Non surv	Liver function	45% 3-year surv32% 3-year non surv
Giannini2000 [30]	Italy	Retrospective	HCV-related HCC	US and AFPevery 6 mo	61(34 S, 27 NS)	NR	NR	NR	68% Surv41% Non surv	None	Median 23-mo surv15-mo non surv
Wong2000 [59]	United States	Retrospective	HCC	US and AFPevery 6–12 mo	91(16 S, 75 NS)	NR	TNMStage I–II	62% Surv45% Non surv	88% Surv41% Non surv	None	65% 3-year surv19% 3-year non surv
Durand1995 [26]	France	Retrospective	HCC	US and AFPevery 6 mo	61(7 S, 54 NS)	NR	Unifocal tumor <3 cm	14% Surv17% Non surv	14% Surv4% Non surv	None	30% 1-year surv35% 1-year non surv
Garcia Gullon1995 [28]	Spain	Retrospective	HCC	USevery 6 mo	99(34 S, 65 NS)	27.3%	Unifocal tumor <5 cm	59% Surv11% Non surv	24% Surv5% Non surv	N/A	NR
Onodera1994 [39]	Japan	Retrospective	HCC	US and AFP	116(19 S, 97 NS)	NR	LCSGJStage I–II	79% Surv31% Non surv	NR	None	57% 3-year surv17% 3-year non surv
Unoura1993 [56]	Japan	Retrospective	HCC	US and AFPevery 3 mo	112(44 S, 68 NS)	NR	NR	NR	NR	None	Median 32-mo surv12-mo non surv
Martinez Cerezo1993 [36]	Spain	Retrospective	HCC	US and AFP	135(43 S, 92 NS)		Unifocal tumor <5 cm	47% Surv15% Non surv	23% Surv8% Non surv	N/A	NR
Tanaka1990 [50]	Japan	Retrospective	HCC	US and AFPevery 3–6 mo	105(22 S, 83 NS)	0%	Unifocal tumor <4 cm	68% Surv23% Non surv	59% Surv33% Non surv	N/A	NR

HR, hazard ratio; LCSGJ, Liver Cancer Study Group of Japan; N/A, not applicable; NR, not reported; NS, non surveillance group; S, surveillance group; US, ultrasound.

Twenty-nine studies reported details regarding the proportion of patients with HCC and underlying cirrhosis. Overall, 90.9% (6,732 of 7,411) of patients had underlying cirrhosis, although rates ranged from 32.4% to 100% between studies. Twenty-seven studies reported information regarding liver function among included patients. The majority (55.5%) of patients (6,018 of 10,853) had Child Pugh A cirrhosis, with higher rates among those who received HCC surveillance (61.3% versus 51.4%, *p*<0.001) (2,607 of 4,255 for surveillance versus 3,213 of 6,247 for non-surveillance). Similarly, patients who received HCC surveillance had lower rates of Child Pugh class C cirrhosis (8.5% versus 11.7%, *p*<0.001) (310 of 3,647 for surveillance versus 592 of 5,062 for non-surveillance).

### Quality Assessment

The quality assessment for included studies is described in Table 2. Out of a maximum 9-point score, 27 studies had quality scores of 5 or 6, 12 studies had a score of 7, and eight had quality scores of 8 or 9. Most studies had appropriate cohort selection, including representativeness of the surveillance cohort and selection of the non-surveillance cohort. All studies ascertained surveillance exposure and HCC outcomes through medical records. However, only six of the 36 studies assessing the impact of surveillance on survival controlled for both lead-time bias and Child Pugh liver function. An additional 13 studies controlled for liver function alone but 17 studies did not control for either factor. Furthermore, 20 studies did not have sufficient follow-up length to assess survival and 27 studies failed to adequately account for patients lost to follow-up.

**Table 2:** Quality assessment of studies.

AuthorYear	Surveillance Cohort Representative	Non-surveillance Cohort Selection	Ascertainment Of Exposure	Outcome Not Initially Present	Control for Potential Confounders^a^	Assessment of Outcome	Follow-up Period	Follow-up of Cohort
Singal2013 [45]	1	1	1	1	0	1	1	1
Wong2013 [60]	1	1	1	1	1	1	0	1
Ayala2012 [18]	1	1	1	1	0	1	0	0
Bouali2012 [20]	1	1	1	1	1	1	0	0
Miguel2012 [37]	1	0	1	1	1	1	1	1
Sarkar2012 [43]	1	1	1	1	1	1	0	0
El-Serag2011 [27]	1	1	1	1	2	1	1	1
Kallwitz2011 [33]	1	1	1	1	0	1	0	0
Reau2011 [41]	1	1	1	1	0	1	1	1
Smirniotopoulos2011 [46]	1	1	1	1	0	1	1	0
Stroffolini2011 [48]	1	1	1	1	1	1	1	1
Yang2011 [61]	1	1	1	1	0	1	0	0
Rodriguez2011 [42]	1	1	1	1	0	1	1	0
Goh2010 [31]	1	1	1	1	0	1	0	1
Jou2010 [32]	1	1	1	1	1	1	1	0
Kuo2010 [34]	1	1	1	1	1	1	1	1
Noda2010 [38]	1	1	1	1	0	1	0	0
Tong2010 [52]	1	1	1	1	1	1	0	0
Tong2010 [53]	1	1	1	1	2	1	0	0
Zapata2010 [63]	1	1	1	1	1	1	1	0
Chan2008 [22]	1	1	1	1	0	1	1	0
Pascual2008 [40]	1	1	1	1	1	1	0	1
Silveira2008 [44]	1	1	1	1	1	1	0	1
Stravitz2008 [47]	1	1	1	1	0	1	0	1
Wong2008 [58]	1	1	1	1	2	1	1	0
Caumes2007 [21]	1	1	1	1	0	1	1	0
Cho2007 [24]	0	1	1	1	1	1	1	0
Davila2007 [25]	1	1	1	1	0	1	1	0
Gellert2007 [29]	1	1	1	1	1	1	1	1
Leykum2007 [35]	1	1	1	1	0	1	0	0
Ando2006 [17]	1	1	1	1	0	1	1	0
Cheung2006 [23]	1	1	1	1	1	1	1	0
Tanaka2006 [49]	1	1	1	1	2	1	1	1
Toyoda2006 [54]	1	1	1	1	1	1	0	0
Taura2005 [51]	1	1	1	1	1	1	1	0
Van Vlierberghe2005 [57]	1	1	1	1	0	1	1	0
Yu2004 [62]	1	1	1	1	2	1	1	1
Trevisani2002 [55]	1	1	1	1	2	1	1	1
Bolondi2001 [19]	1	1	1	1	1	1	0	0
Giannini2000 [30]	1	1	1	1	0	1	0	0
Wong2000 [59]	1	1	1	1	0	1	0	0
Durand1995 [26]	1	1	1	1	0	1	1	0
Garcia Gullon1995 [28]	1	1	1	1	0	1	1	0
Onodera1994 [39]	1	1	1	1	0	1	0	0
Unoura1993 [56]	1	1	1	1	0	1	0	0
Martinez Cerezo1993 [36]	1	1	1	1	0	1	1	0
Tanaka1990 [50]	0	1	1	1	0	1	1	0

a Confounders of interest were lead-time bias and liver function for survival, liver function, and performance status for treatment eligibility, and liver function and body mass index for early stage tumor detection.

### Association between HCC Surveillance and Detection of Tumors at an Early Stage

Thirty-eight studies, with a total of 10,904 patients, included data on tumor stage stratified by receipt of HCC surveillance [17],[18],[20],[21],[23]–[26],[28],[29],[31]–[50],[52]–[55],[57],[59],[61],[63]. Twenty-four studies defined early stage using BCLC or Milan criteria [17],[18],[24],[29],[32]–[35],[37],[38],[41]–[45],[47]–[49],[52],[53],[55],[57],[61],[63], whereas six studies used other staging systems (e.g., tumor node metastases [TNM]) [23],[31],[39],[46],[54],[59], and eight used operational definitions (e.g., unifocal lesion less than 3 cm) [20],[21],[25],[26],[28],[36],[40],[50] (Table 1). The 24 studies using BCLC or Milan criteria included a total of 6,573 patients, of whom 2,815 (44.1%) were diagnosed by surveillance.

**Figure 2:**
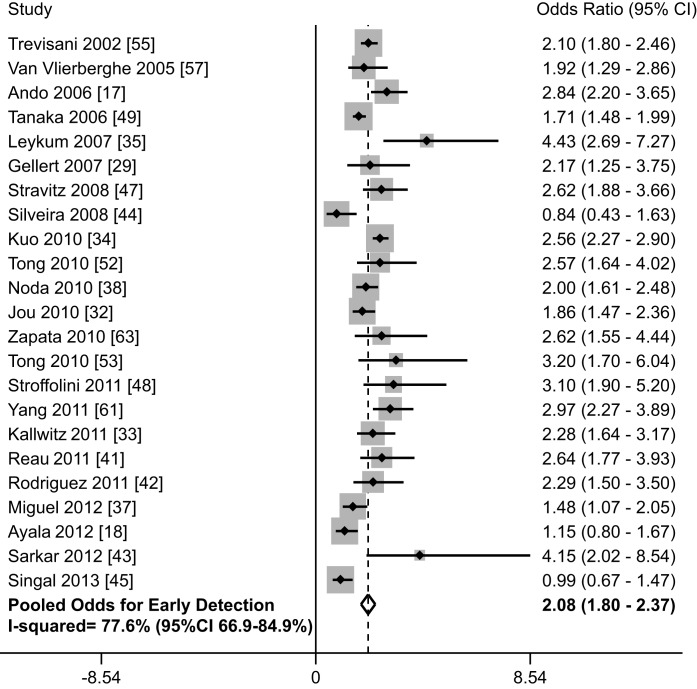
Association between HCC surveillance and early tumor detection rates.

When including all 38 studies, patients who underwent surveillance were significantly more likely to be found at an early stage (OR 2.11, 95% CI 1.88–2.33); however, there was significant heterogeneity (I^2^ = 73%, *p*<0.001) (Table 1). When only including studies using BCLC or Milan criteria, there was little change in effect size (OR 2.08, 95% CI 1.80–2.37) or heterogeneity (I^2^ = 77%, *p*<0.001). On subset analysis of the six studies using BCLC to define early stage, the pooled odds ratio was also stable at 1.96 (95% CI 1.41–2.73) [18],[24],[32],[34],[37],[42]. One notable outlier was a study by Cho and colleagues, which had a relative risk of 37.81 (95% CI 5.27–271.13) [24]. Only data on patients younger than 30 years old were reported for this study, so we excluded it from further analyses. Heterogeneity (I^2^ = 78%, *p*<0.001) could not be improved with removal of additional studies, and meta-influence analysis did not suggest undue influence of any single study. Among the 23 remaining studies, HCC surveillance was significantly associated with early stage tumor detection (OR 2.08, 95% CI 1.80–2.37) (Figure 2). The pooled rate of early stage HCC among patients undergoing surveillance was 70.9% (95% CI 69.3%–72.6%) (2,047 of 2,885 patients), compared to only 29.9% (95% CI 28.4–31.4%) (1,034 of 3,463 patients) among those who presented symptomatically and/or diagnosed incidentally.

We performed pre-planned subset analyses according to study design, location of study, study period, and type of surveillance tests used (Table 4). Rates of early tumor detection were consistent across study location (OR 2.22 [95% CI 1.75–2.81] among studies conducted in Asia [17],[34],[38],[49] versus 2.00 [95% CI 1.70–2.35] among studies in Europe [37],[42],[55],[57],[63] versus 2.31 [95% CI 1.79–2.99] among studies in the United States [32],[33],[35],[41],[43],[44],[45],[47],[52],[53],[61]), study period (OR 2.22 [95% CI 1.77–2.79] among studies assessing surveillance in the 1990s [17],[29],[49],[53],[55] versus 2.18 [95% CI 1.86–2.56] among studies assessing surveillance after 2000 [18],[32]–[35],[37],[38],[41]–[43],[45],[47],[52],[57],[61],[63]), and type of surveillance tests (OR 2.04 [95% CI 1.55–2.68] with ultrasound alone [18],[32],[38],[47],[61] versus 2.16 [95% CI 1.80–2.60] with ultrasound and/or AFP [17],[29],[34],[35],[37],[42]–[45],[49],[52],[53],[55],[63]). There was no significant difference in the association between HCC surveillance and early stage tumor detection by study design (*p* = 0.10), with patients detected by surveillance being more likely to be found at an early stage in both subgroups. The pooled odds ratio was 2.30 (95% CI 1.98–2.67) among retrospective studies [17],[18],[29],[32]–[35],[38],[41],[43],[44],[47],[49],[52],[53],[61],[63], compared to 1.70 (95% CI 1.29–2.26) among studies in which data were prospectively collected [37],[42],[45],[55],[57]. Heterogeneity in early tumor detection may be related to several factors including variations in ultrasound operator experience and technique, patient body habitus, and liver nodularity, which we were unable to explore given the lack of patient-level data.

### Association between HCC Surveillance and Receipt of Curative Treatment

**Figure 3:**
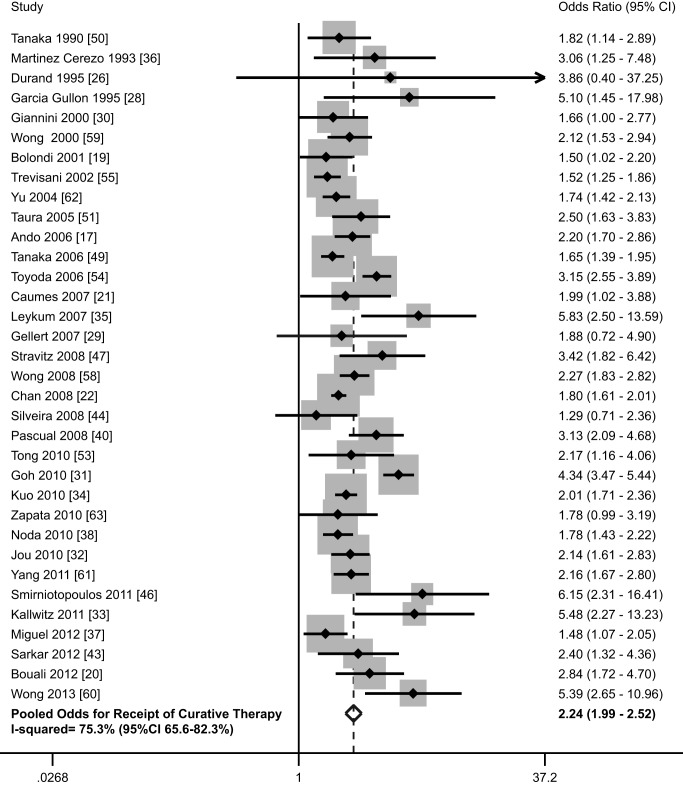
Association between HCC surveillance and curative treatment rates.

Thirty-four studies, with a total of 12,187 patients, assessed the association of HCC surveillance with receipt of curative therapy [17],[19],[20]–[22],[26],[28]–[38],[40],[43],[44],[46],[47],[49],[50],[51],[53]–[55],[58]–[63]. Of the included patients, 4,655 (38.2%) were detected by surveillance and 7,532 (61.8%) presented symptomatically or were diagnosed incidentally. Patients diagnosed by surveillance were significantly more likely to undergo curative therapy, with a pooled odds ratio of 2.24 (95% CI 1.99–2.52) (Figure 3; Table 1). We found heterogeneity among studies (I^2^ = 75.3%, *p*<0.001). Meta-influence analysis did not suggest undue influence of any single study. Although four studies [33],[35],[46],[60] appeared to be outliers, we did not find clinical heterogeneity justifying their exclusion. The association between HCC surveillance and receipt of curative therapy did not substantially change if these four studies had been excluded (OR 2.11, 95% CI 1.89–2.37). The pooled rate of curative treatment receipt among patients undergoing surveillance was 51.6% (95% CI 50.2–53.0%) (2,402 of 4,655), compared to only 23.7% (95% CI 22.8%–24.7%) (1,790 of 7,532) among those who presented symptomatically or were diagnosed incidentally.

Among the 16 cohort studies that reported both early detection (using Milan or BCLC criteria) and curative treatment rates [17],[29],[32]–[35],[37],[38],[43],[44],[47],[49],[53],[55],[61],[63], we found a moderately strong positive correlation between early detection rates and curative treatment rates between studies (Pearson's correlation r = 0.54) (Figure S4). This finding suggests the association between surveillance and receipt of curative treatment is mediated by improved early tumor detection rates.

We performed pre-planned subset analyses, according to study design, location of study, study period, and type of surveillance tests used (Table 4). Rates of curative therapy receipt were consistent across study design (OR 2.18 [95% CI 1.94–2.45] among retrospective studies [17],[19],[20],[26],[28]–[30],[32]–[36],[38],[43],[44],[46],[47],[49],[50],[51],[53]–[55],[58]–[63] versus 2.37 [95% CI 1.51–3.72] among prospective studies [21],[22],[31],[37],[40]), study period (OR 2.12 [95% CI 1.25–3.61] among studies assessing surveillance prior to 1990 [26],[44],[50],[54] versus 2.23 [95% CI 1.87–2.67] among studies assessing surveillance in the 1990s [17],[19],[20], versus 2.13 [95% CI 1.85–2.44] among studies assessing surveillance after 2000 [21],[32],[34],[35],[37],[38],[43],[46],[47],[58],[61],[63]), and type of surveillance tests (OR 2.23 [95% CI 1.83–2.71] with ultrasound alone [20],[28],[32],[38],[46],[47],[61],[62] versus 2.19 [95% CI 1.89–2.53] with ultrasound and/or AFP [17],[19],[22],[26],[29]–[31],[34]–[37],[40],[43],[44],[49],[50],[51],[53]–[55],[58]–[60],[63]). Finally, there was no significant difference in the strength of association between HCC surveillance and curative therapy receipt by study location (*p* = 0.20); patients detected by surveillance were significantly more likely to receive curative therapy in both subgroups. The pooled odds of curative therapy were 1.87 (95% CI 1.51–2.31) for studies conducted in Europe [19],[21],[26],[28],[30],[36],[37],[40],[55],[63], 2.19 (95% CI 1.84–2.61) for studies conducted in Asia [17],[22],[31],[34],[38],[49],[50],[51],[54],[58],[62], and 2.52 (95% CI 1.99–3.20) for studies conducted in the United States [32],[33],[35],[43],[44],[46],[47],[53],[59],[61].

### Association between HCC Surveillance and Overall Survival

**Figure 4:**
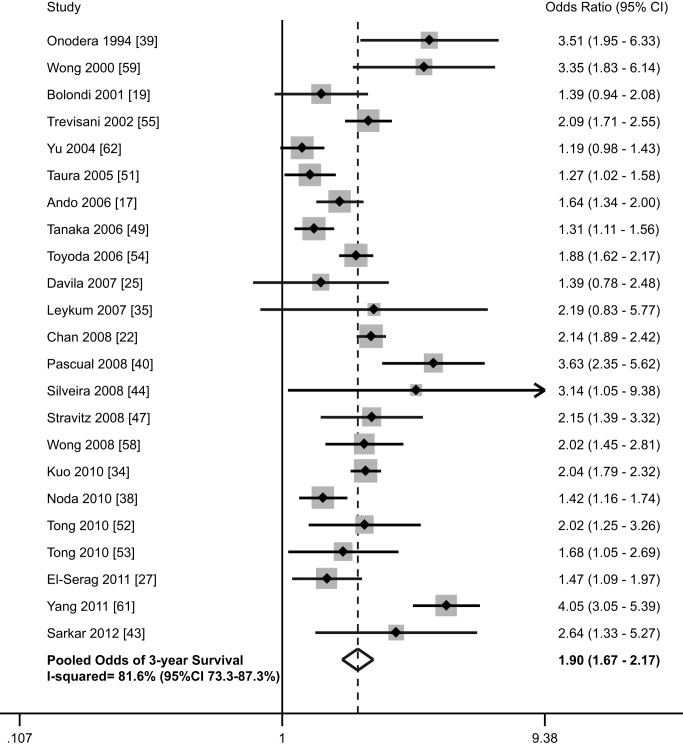
Association between HCC surveillance and survival.

Thirty-six studies, with a total of 13,361 patients (40.9% [*n* = 5,466] detected via surveillance), included data on survival stratified by receipt of HCC surveillance [17]–[20],[22]–[27],[29]–[31],[33]–[35],[37]–[40],[43],[44],[47],[49],[51]–[62]. There was substantial variability in reporting of survival data, with several studies reporting 1-year and/or 3-year survival rates, some reporting median survival without confidence intervals, and others showing a Kaplan Meier curve (Table 1). The most commonly reported survival outcome was 3-year survival, so this was used for further analysis. Three-year survival rates were estimated from Kaplan Meier curves if data were not otherwise presented. Among these 23 studies, HCC surveillance was significantly associated with improved survival, with a pooled odds ratio of 1.90 (95% CI 1.67–2.17) (Figure 4) [17],[19],[22],[25],[27],[34],[35],[38]–[40],[43],[44],[47],[49],[51]–[55],[58],[59],[61],[62]. The pooled 3-year survival rate was 50.8% among the 4,735 patients who underwent HCC surveillance, compared to only 27.9% among the 6,115 patients without prior surveillance (*p*<0.001).

We performed pre-planned subset analyses, according to location of study, study period, proportion of Child Pugh C cirrhosis, and study quality (Table 4). The pooled 3-year survival rates for patients with and without surveillance were the highest among studies conducted in Asia [17],[22],[34],[38],[39],[49],[51],[54],[58],[62] (57.4% and 31.7%, respectively) (1,693 of 2,947 for surveillance versus 1,340 of 4,233 for non-surveillance), intermediate among studies from Europe [19],[40],[55] (47.3% and 21.8%, respectively) (259 of 548 for surveillance versus 159 of 728 for non-surveillance), and the lowest among studies conducted in the United States [25],[27],[35],[43],[44],[47],[52],[53],[59],[61] (36.5% and 18.2%, respectively) (453 of 1,240 for surveillance versus 210 of 1,154 for non-surveillance). Pooled 3-year survival rates were 51.1% (555/1086) and 25.4% (179/704) for surveillance and non-surveillance groups among studies assessing surveillance prior to 1990 [39],[44],[54], 57.6% (1,122/1,947) and 32.2% (893/2,773), respectively, among studies assessing surveillance during the 1990s [17],[19],[22],[40],[49],[51],[53],[55],[59], and 42.8% (728/1,702) and 24.1% (637/2,638) among those assessing surveillance after 2000 [25],[27],[34],[35],[38],[43],[47],[52],[58],[61]. There were 15 studies reporting the proportion of Child C patients and data regarding 3-year survival rates [19],[22],[25],[34],[40],[44],[47],[49],[51]–[55],[58],[61]. As anticipated, 3-year survival rates were inversely related to the proportion of patients with Child C cirrhosis. The pooled 3-year survival rates were 57.0% (1,033 of 1,813 patients) and 29.2% (960 of 3,293 patients) in patients with and without surveillance, respectively, among the eight studies with less than 10% Child Pugh C patients 22,[34],[49],[51]–[53],[55],[58]. In the seven studies with more than 10% Child Pugh C patients, the 3-year survival rates were only 49.8% (795 of 1,597 patients) and 22.0% (311 of 1,411 patients), respectively [19],[25],[40],[44],[47],[54],[61]. Finally, we evaluated survival according to study quality, with high-quality studies defined as those with a score of 7–9 [27],[34],[40],[44],[49],[51],[53],[55],[58],[62] and low-quality studies defined as those with scores less than 7 [17],[19],[22],[25],[35],[38],[39],[43],[47],[52],[54],[59],[61]. High-quality and low-quality studies had similar 3-year survival rates in the non-surveillance groups (28.8% versus 26.9%, respectively, *p* = 0.09) (965 of 3,346 patients for high-quality studies and 744 of 2,769 patients for low-quality studies). However, 3-year survival rates were significantly lower in the surveillance groups in high quality studies than low-quality studies (45.6% versus 54.7%, respectively, *p*<0.001) (927 of 2,031 patients for high-quality studies versus 1,478 of 2,704 patients for low-quality studies).

Six studies evaluated any potential benefit of surveillance on survival, after adjusting for lead-time bias (Table 3) [27],[49],[53],[55],[58],[62]. Among these studies, HCC surveillance was still associated with a significant improvement in survival (3-year survival rates 39.7% versus 29.1%, *p*<0.001) (556 of 1,401 patients for surveillance versus 567 of 1,946 for non-surveillance) (*p*<0.001). El-Serag and colleagues reported improved survival when assuming a tumor doubling time of 70 days (OR 0.81, 95% CI 0.70–0.94) [27]. Assuming a tumor doubling time of 90 days, surveillance was associated with improved survival in studies by Wong (*p* = 0.04) [58] and Tanaka (*p* = 0.02) [49]. Tong and colleagues found significantly improved 3-year survival (62.5% versus 36.6%, *p* = 0.007) after adjusting for a lead-time of 3.9 months, which was based on tumor doubling time among their patients [53]. Yu and colleagues also found significantly reduced mortality at 3 years among those with surveillance (OR 0.35, 95% CI 0.24–0.49) [62]. Adjusting for lead-time bias (239 days for 6-month surveillance and 98 days for annual surveillance), Trevisani and colleagues found patients undergoing surveillance had a median survival of 30 months, which was significantly better than the 20-month median survival among patients with incidentally discovered tumors (*p*<0.001) or the 9-month median survival among patients who presented symptomatically (*p*<0.001) [55].

**Table 3:** Studies assessing survival benefit of surveillance after adjusting for lead time bias.

AuthorYear	Tumor Doubling Time	Estimated Lead Time	Survival Rates	Statistical Significance
El-Serag2011 [27]	70 days	70 days	Median survival298 vs. 130 days	OR 0.81(95% CI 0.70–0.94)
Tong2010 [53]	216 days	118 days	3-year survival62.5% vs. 36.6%	*p* = 0.007
Wong2008 [58]	90 days	236 days	2-year survival49.4% vs. 28.6%	*p* = 0.035
Tanaka2006 [49]	90 days	238 days	Median survival6.3 vs. 5.3 years^a^	*p* = 0.016
Yu2004 [62]	Not reported	Not reported	3-year survival49.0% vs. 41.2%^a^	OR 0.35(95% CI 0.24–0.49)
Trevisani2002 [55]	Not reported	98–239 days	Median survival30 vs. 20 mo.	*p*<0.001

a Estimated from Kaplan Meier curve.

**Table 4:** Subgroup analyses for association between HCC surveillance and early detection, curative treatment rates, and survival.

Variable	Subgroup	Odds Ratio
**Early detection**		
Study design	Prospective [37],[42],[45],[55],[57]	OR 1.70 (95% CI 1.29–2.26)
	Retrospective [17],[18],[29],[32]–[35],[38],[41],[43],[44],[47],[49],[52],[53],[61],[63]	OR 2.30 (95% CI 1.98–2.67)
Location of study	Asia [17],[34],[38],[49]	OR 2.22 (95% CI 1.75–2.81)
	Europe [37],[42],[55],[57],[63]	OR 2.00 (95% CI 1.70–2.35)
	United States [32],[33],[35],[41],[43]–[45],[47],[52],[53],[61]	OR 2.31 (95% CI 1.79–2.99)
Study period	During 1990s [17],[29],[49],[53],[55]	OR 2.22 (95% CI 1.77–2.79)
	After 2000 [18],[32]–[35],[37],[38],[41]–[43],[45],[47],[52],[57],[61],[63]	OR 2.18 (95% CI 1.86–2.56)
Type of surveillance test	Ultrasound alone [18],[32],[38],[47],[61]	OR 2.04 (95% CI 1.55–2.68)
	Ultrasound ± AFP [17],[29],[34],[35],[37],[42]–[45],[49],[52],[53],[55],[63]	OR 2.16 (95% CI 1.80–2.60)
Study size	More than 100 patients [17],[18],[29],[32]–[34],[37],[38],[41],[42],[47],[48],[50],[52],[55],[57],[61]	OR 2.13 (95% CI 1.88–2.39)
**Receipt of curative treatment**		
Study design	Prospective [21],[22],[31],[37],[40]	OR 2.37 (95% CI 1.51–3.72)
	Retrospective [17],[19],[20],[26],[28]–[30],[32]–[36],[38],[43],[44],[46],[47],[49]–[51],[53]–[55],[58]–[63]	OR 2.18 (95% CI 1.94–2.45)
Location of study	Asia [17],[22],[31],[34],[38],[49]–[51],[54],[58],[62]	OR 2.19 (95% CI 1.84–2.61)
	Europe [19],[21],[26],[28],[30],[36],[37],[40],[55],[63]	OR 1.87 (95% CI 1.51–2.31)
	United States [32],[33],[35],[43],[44],[46],[47],[53],[59],[61]	OR 2.52 (95% CI 1.99–3.20)
Study period	Prior to 1990 [26],[44],[50],[54]	OR 2.12 (95% CI 1.25–3.61)
	During 1990s [17],[19],[20],[22],[28]–[31],[36],[40],[49],[51],[53],[55],[59],[60],[62]	OR 2.23 (95% CI 1.87–2.67)
	After 2000 [21],[32],[34],[35],[37],[38],[43]–[47],[58],[61],[63]	OR 2.13 (95% CI 1.85–2.44)
Type of surveillance test	Ultrasound alone [20],[28],[32],[38],[46],[47],[61],[62]	OR 2.23 (95% CI 1.83–2.71)
	Ultrasound ± AFP [17],[19],[22],[26],[29]–[31],[34]–[37],[40],[43],[44],[49]–[51],[53]–[55],[58]–[60],[63]	OR 2.19 (95% CI 1.89–2.53)
Study size	More than 100 patients [17],[19]–[22],[29],[31]–[34],[36]–[38],[40],[47],[49]–[51],[54],[55],[58],[61],[62]	OR 2.18 (95% CI 1.91–2.48)
**3-year survival**		
Location of study	Asia [17],[22],[34],[38],[39],[49],[51],[54],[58],[62]	57.4% for surveillance vs. 31.7% for non-surveillance
	Europe [19],[40],[55]	47.3% for surveillance vs. 21.8% for non-surveillance
	United States [25],[27],[35],[43],[44],[47],[52],[53],[59],[61]	36.5% for surveillance vs. 18.2% for non-surveillance
Study period	Prior to 1990 [39],[44],[54]	51.1% for surveillance vs. 25.4% for non-surveillance
	During 1990s [17],[19],[22],[40],[49],[51],[53],[55],[59]	57.6% for surveillance vs. 32.2% for non-surveillance
	After 2000 [25],[27],[34],[35],[38],[43],[47],[52],[58],[61]	42.8% for surveillance vs. 24.1% for non-surveillance
Liver function	Child C cirrhosis ≥10% cohort [19],[25],[40],[44],[47],[54],[61]	57.0% for surveillance vs. 29.2% for non-surveillance
	Child C cirrhosis <10% cohort [22],[34],[49],[51],[52],[53],[55],[58]	49.8% for surveillance vs. 22.0% for non-surveillance
Overall study quality	Low quality [17],[19],[22],[25],[35],[38],[39],[43],[47],[52],[54],[59],[61]	54.7% for surveillance vs. 26.9% for non-surveillance
	High quality [27],[34],[40],[44],[49],[51],[53],[55],[58],[62]	45.6% for surveillance vs. 28.8% for non-surveillance
Lead time bias assessment	Did not adjust for lead time bias [17],[19],[22],[25],[34],[35],[38]–[40],[43],[44],[47],[51],[52],[54],[59],[61]	55.5% for surveillance vs. 27.4% for non-surveillance
	Adjusted for lead time bias [27],[49],[53],[55],[58],[62]	39.7% for surveillance vs. 29.1% for non-surveillance
Study size	More than 100 patients [17],[19],[22],[25],[27],[34],[38]–[40],[47],[49],[51],[52],[54],[55],[58],[61],[62]	50.7% for surveillance vs. 39.0% for non-surveillance

## Discussion

To the best of our knowledge, our meta-analysis is the first to critically examine available literature and characterize the potential impact of HCC surveillance on outcomes in patients with cirrhosis. We demonstrated HCC surveillance was associated with significant improvement in early tumor detection and receipt of curative therapies. Most importantly, HCC surveillance was associated with a significant improvement in overall survival. However, there are limitations in current literature, including many studies having insufficient duration of follow-up to adequately assess survival and the majority not adjusting for liver function or lead-time bias. Overall, in the absence of randomized data of surveillance efficacy, our meta-analysis provides sufficient evidence to support guidelines that recommend HCC surveillance in patients with cirrhosis.

The lack of randomized data supporting HCC surveillance in cirrhotic patients has caused some providers to question its benefit, which may contribute to low utilization rates. Prior studies have reported HCC surveillance rates below 20% in the United States, with lower rates among primary care physicians than gastroenterologists/hepatologists [64]–[68]. However, a lack of randomized data does not necessarily equate to a lack of efficacy. For example, colonoscopy is widely embraced for colorectal cancer screening, without randomized data, based on cohort and case-control studies as well as extrapolation of fecal occult blood test data [69]–[71]. HCC surveillance fulfills all criteria established by the World Health Organization for a surveillance program [72]: the disease burden of HCC is an important health problem, there is an identifiable target population, surveillance is accepted by patients and providers, surveillance achieves an acceptable level of accuracy, there are standardized recall procedures, surveillance is affordable, there is an advantage of treating occult HCC, and surveillance reduces mortality. Our meta-analysis highlights consistent improvements in early tumor detection, receipt of curative therapy, and overall survival with HCC surveillance among patients with cirrhosis. In light of these data, a randomized controlled trial of HCC surveillance could be deemed unethical. In fact, prior attempts at a randomized trial were unsuccessful, as patients refused participation and desired surveillance after the benefits and harms were discussed [73].

We found substantial statistical heterogeneity between studies, suggesting benefits of surveillance may not be uniform among all patients. Several studies included patients with Child C cirrhosis, which may explain some heterogeneity with regard to treatment eligibility and survival. Trevisani and colleagues demonstrated the survival benefit of HCC surveillance was most marked in patients with Child A cirrhosis [55]. Those with Child C cirrhosis failed to achieve a significant benefit, given lower treatment eligibility rates and higher competing risk of liver-related mortality. Surveillance is not recommended in patients with Child C cirrhosis unless they are transplant candidates [5], so their inclusion in several studies may have mitigated reported benefits of surveillance on treatment eligibility and overall survival.

Furthermore, the risk of HCC may not be uniform across patients and etiologies of liver disease [74]. For example, patients with HCV cirrhosis have a higher risk of HCC than those with alcohol-induced cirrhosis or NASH [75],[76]. Predictive models have been created using several risk factors but are limited by moderate accuracy to date [77],[78]. Similarly, surveillance is performed with ultrasound and AFP in all patients despite variations in accuracy among patients. Ultrasound is less sensitive in obese patients and those with advanced fibrosis, whereas AFP may be less accurate among HCV positive patients [8],[79]. Accurate assessment of HCC risk and surveillance performance characteristics may allow personalized surveillance programs, which could optimize benefits and cost-effectiveness of HCC surveillance. Surveillance may be avoided in low-risk patients, whereas high-risk patients could benefit from a more intensive surveillance regimen.

On subgroup analysis for the association between HCC surveillance and overall survival, we found substantial differences according to study location. We did not find any significant variation in study quality (*p* = 0.37) or size (*p* = 0.07) by study location that might help explain the differences. Further studies are needed to explore this heterogeneity, as there are several potential explanations. There are differences in patient populations, such as higher rates of obesity and NASH-related cirrhosis in the United States than Europe and Asia [80], which may affect treatment response and recurrence rates. There are also differential rates and choice of curative treatment among patients found at an early stage, which can influence response rates, recurrence rates, and overall survival [81].

Results from our study must be interpreted within the limitations of included studies. Many studies failed to adequately account for patients lost to follow-up and did not have sufficient follow-up to adequately assess survival. Furthermore, several studies used operational definitions of surveillance, such as ultrasound or AFP in a two-year period, which were not consistent with guideline recommendations. Guidelines recommend ultrasound every 6 months to optimize sensitivity, and AFP should not be used alone without imaging [5]. Clear definitions and measures should be used in future studies to better interpret and quantify any benefits of HCC surveillance.

All studies in this meta-analysis were non-randomized cohort studies, with potential for lead-time and length-time biases. However, several studies demonstrated a significant improvement in survival after statistically adjusting for lead-time bias [82]. Furthermore, lead-time bias may be less problematic for patients diagnosed at an early stage by surveillance, given the selective availability of curative options at that stage. Liver transplantation, surgical resection, and RFA have been associated with 5-year survival rates approaching 70% but are only available for patients with early stage tumors [5]. The survival benefit of HCC surveillance is contingent on subsequent receipt of curative therapy [83]. This relationship is further highlighted by the strong positive correlation between early tumor detection and curative treatment rates among studies in our meta-analysis.

Study results were also potentially limited by selection bias, with a differential distribution of liver function and/or performance status among surveillance and non-surveillance groups. Surveillance group patients were less likely to have Child Pugh C liver disease, although liver function was not reported in all studies. Other studies have suggested that patients with hepatic decompensation are more likely to have recognized cirrhosis and therefore receive surveillance [68]. We did not find information regarding functional status in any of the included studies. Detailed reporting of performance status and liver function is important given both are key factors in determining treatment eligibility. Patients with poor functional status or Child C cirrhosis, if not transplant candidates, should be excluded given HCC surveillance is not recommended in these subgroups. Finally, a comprehensive assessment of surveillance should weigh benefits and harms; however, no study in our meta-analysis assessed downstream harms. Although ultrasound and AFP have minimal direct harms, there are potential downstream harms from recall policies (e.g., complications of liver biopsy or cross-sectional imaging) that should be considered in future studies.

In summary, current data suggest that HCC surveillance is associated with significant improvement in early tumor detection. By facilitating receipt of curative therapy in a higher proportion of patients, HCC surveillance is associated with a significant improvement in overall survival. There are notable limitations in current literature, including many studies failing to adequately adjust for lead-time bias. However, the preponderance of data that consistently demonstrate benefits should provide sufficient rationale to recommend HCC surveillance, even in the absence of a randomized controlled trial among patients with cirrhosis.

## Supporting Information

Figure S1 **Funnel plot for HCC surveillance and early detection.**
(EPS)

Figure S2 **Funnel plot for HCC surveillance and receipt of curative treatment.**
(EPS)

Figure S3 **Funnel plot for HCC surveillance and survival.**
(EPS)

Figure S4 **Association between early detection by HCC surveillance and receipt of curative treatment.**
(EPS)

Table S1 **MOOSE checklist.**
(DOC)

## Abbreviations

AFP: alpha fetoprotein
BCLC: Barcelona Clinic Liver Cancer
HBV: hepatitis B virus
HCC: hepatocellular carcinoma
HCV: hepatitis C virus
NASH: nonalcoholic steatohepatitis
OR: odds ratio
RFA: radiofrequency ablation
TNM: tumor node metastases
