# Supplementary material for: Early Detection, Curative Treatment, and Survival Rates for Hepatocellular Carcinoma Surveillance in Patients with Cirrhosis: A Meta-analysis
Source: PLoS Med. 2014 Apr 1;11(4):e1001624. doi: 10.1371/journal.pmed.1001624 (PMC3972088; doi:10.1371/journal.pmed.1001624)
Supplement: Table S1 — MOOSE checklist. (DOC) [file pmed.1001624.s005.doc]

**MOOSE Checklist**

| **Criteria** | | **Brief description of how the criteria were handled in the meta-analysis** |
| --- | --- | --- |
| **Reporting of background should include** | |  |
| √ | Problem definition | Given the lack of a randomized trial of HCC surveillance among patients with cirrhosis, a meta-analysis of cohort and case-control studies can serve to better characterize any potential benefits of HCC surveillance. (top page 5) |
| √ | Hypothesis statement | HCC surveillance detects HCC at an early stage, when it is amenable to curative therapy, and reduces mortality. (bottom page 4) |
| √ | Description of study outcomes | Detection of tumors at an early stage,  Receipt of curative therapies, and  Overall survival (top page 5 and page 6, study selection) |
| √ | Type of exposure or intervention used | HCC surveillance (top page 5 and page 6, study selection) |
| √ | Type of study designs used | We included case-control studies, prospective cohort studies, and cross-sectional studies. There have not been any randomized controlled studies in this population (top page 5) |
| √ | Study population | Patients with cirrhosis (top page 5 and page 6, study selection) |
| **Reporting of search strategy should include** | |  |
| √ | Qualifications of searchers | The credentials of the two investigators AS and AP are indicated in the author list (page 1). |
| √ | Search strategy, including time period included in the synthesis and keywords | Ovid interface to Medline from January 1, 1989 to January 1, 2014  National meeting abstracts from 2010 – 2012  (page 5, data sources and searches) |
| √ | Databases and registries searched | Ovid interface to Medline (page 5, data sources and searches) |
| √ | Search software used, name and version, including special features | Ovid interface to Medline (page 5, data sources and searches) |
| √ | Use of hand searching | We hand-searched annual meeting abstracts and bibliographies of studies (recursive literature search)  (page 5, data sources and searches) |
| √ | List of citations located and those excluded, including justifications | Details of the literature search process are outlined in the search map. The citation list is available upon request  (Figure 1) |
| √ | Method of addressing articles published in languages other than English | We included studies published in English or Spanish  (page 6, study selection) |
| √ | Method of handling abstracts and unpublished studies | We searched national meeting abstracts over past 3 years. We also consulted with expert hepatologists to identify additional references or unpublished data. Authors were contacted as needed. (page 5, data sources and searches) |
| √ | Description of any contact with authors | We planned to contact authors for missing data as needed; however, this was not necessary for any studies that appeared relevant. (not described in manuscript as was not needed) |
| **Reporting of methods should include** | |  |
| √ | Description of relevance or appropriateness of studies assembled for assessing the hypothesis to be tested | Detailed inclusion and exclusion criteria were described in the methods section. (page 6, study selection) |
| √ | Rationale for the selection and coding of data | Data extracted from each of the studies were relevant to the population characteristics, study design, exposure, and outcomes. (page 7, data extraction) |
| √ | Assessment of confounding | We reported differences in liver function between the two groups (surveillance and non-surveillance). We performed subset analyses according to several variables including study location, study period, and liver function. (bottom page 8) |
| √ | Assessment of study quality, including blinding of quality assessors; stratification or regression on possible predictors of study results | Two investigators assessed study quality by a modified checklist based upon the Ottawa-Newcastle scale. We performed subgroup analysis based on quality score for overall survival outcome. (page 7, quality assessment) |
| √ | Assessment of heterogeneity | Heterogeneity was evaluated graphically by examination of forest plots and then statistically by the chi-squared test of heterogeneity and the inconsistency index (middle page 8) |
| √ | Description of statistical methods in sufficient detail to be replicated | Description of methods of meta-analyses, sensitivity analyses, and quality assessment are detailed in the methods. (page 7-8) |
| √ | Provision of appropriate tables and graphics | We included search map, characteristics of studies, and forest plots. |
| **Reporting of results should include** | |  |
| √ | Graph summarizing individual study estimates and overall estimate | Figures 2, 3, and 4 |
| √ | Table giving descriptive information for each study included | Table 1 |
| √ | Results of sensitivity testing | This is detailed in results (pages 10-11, pages 12-13, and pages 14-15) |
| √ | Indication of statistical uncertainty of findings | 95% confidence intervals were presented with all summary estimates, I^2^ values and results of sensitivity analyses |
| **Reporting of discussion should include** | |  |
| √ | Quantitative assessment of bias | Sensitivity analyses indicate heterogeneity in strengths of the association due to most common biases, including lead-time bias (Table 2 and page 18). |
| √ | Justification for exclusion | We justified exclusion criteria of outliers for clinical reasons (e.g. Cho excluded because this only included patients younger than 30 years old). Studies without clinical reasons for exclusion were not excluded. (page 12) |
| √ | Assessment of quality of included studies | We discussed results of quality assessment and impact on results of meta-analysis (page 15, pages 18-19). |
| **Reporting of conclusions should include** | |  |
| √ | Consideration of alternative explanations for observed results | We discussed limitations of current literature including lead-time and length-time biases. (pages 18-19) |
| √ | Generalization of the conclusions | We noted that our results should only be applied to patients with intact liver function and performance status. (top page 20) |
| √ | Guidelines for future research | We recommend future studies adjust for known biases, including lead-time bias, and selection bias (e.g. differences in liver function). We also recommend studies are needed to evaluate potential harms of surveillance. (page 19-20) |
| √ | Disclosure of funding source | Amit Singal’s work is supported by the Center for Translational Medicine, NIH/NCATS Grant KL2 TR000453, NIH/NCATS Grant UL1-TR000451, and the ACG Junior Faculty Development Award. However, no specific funding for this project was obtained. (page 27) |
